# Loss of E-cadherin expression is not a prerequisite for c-erbB2-induced epithelial-mesenchymal transition

**DOI:** 10.3892/ijo.2014.2424

**Published:** 2014-05-07

**Authors:** GISELA M.A. NILSSON, NOREEN AKHTAR, MARIE KANNIUS-JANSON, DAN BAECKSTRÖM

**Affiliations:** 1 Department of Medical Biochemistry and Cell Biology, Institute of Biomedicine, University of Gothenburg, Gothenburg, Sweden; 2 Department of Chemistry and Molecular Biology, University of Gothenburg, Gothenburg, Sweden

**Keywords:** mammary epithelium, breast cancer, vimentin, epidermal growth factor receptor family, cell density, cell-cell adhesion

## Abstract

Recent research into the mechanisms of tumour cell invasiveness has highlighted the parallels between carcinogenesis and epithelial-mesenchymal transition (EMT), originally described as a developmental transdifferentiation program but also implicated in fibrosis and cancer. In a model system for mammary carcinogenesis, we previously observed that induced signalling from a homodimer of the c-erbB2 (HER2) receptor tyrosine kinase in an initially non-malignant mammary cell line caused EMT where i) cell scattering occurred before downregulation of the cell-cell adhesion molecule E-cadherin and ii) the progress of EMT was dramatically delayed when cells were grown at high density. Here, we have further analysed these phenomena. Ectopic expression of E-cadherin concomitant with c-erbB2 signalling was unable to impede the progression of EMT, suggesting that E-cadherin downregulation is not required for EMT. Furthermore, fibroblast-like cells isolated after EMT induced in the presence or absence of ectopic E-cadherin expression showed highly similar morphology and vimentin expression. E-cadherin expressed in these fibroblastic cells had a subcellular localisation similar to that found in epithelial cells, but it exhibited a much weaker attachment to the cytoskeleton, suggesting cytoskeletal rearrangements as an important mechanism in EMT-associated cell scattering. We also investigated whether density-dependent inhibition of EMT is mediated by E-cadherin as a sensor for cell-cell contact, by expressing dominant-negative E-cadherin. While expression of this mutant weakened cell-cell adhesion, it failed to facilitate EMT at high cell densities. These results indicate that loss of E-cadherin expression is a consequence rather than a cause of c-erbB2-induced EMT and that density-dependent inhibition of EMT is not mediated by E-cadherin signalling.

## Introduction

Epithelial-mesenchymal transition (EMT) is a crucially important phenotypic conversion in which epithelial cells dissociate from each other, largely lose cell-cell adhesion and acquire a migratory, fibroblast-like phenotype (1). EMT occurs at various stages of embryonal development, including such early events as the generation of mesoderm and the formation of the neural crest. Typically, EMT entails the disassembly of epithelial cell-cell junctions such as adherens junctions, tight junctions and desmosomes, as well as a radical reorganisation of the cytoskeleton. Cells lose basolateral-apical polarity and instead acquire a leading edge-trailing edge polarity. At the level of gene expression, genes coding for components of cell-cell junctions become inactive, as well as those of certain intermediate filament proteins, which are replaced by others. Typical markers for EMT are loss of E-cadherin, the cell-cell adhesion molecule of epithelial adherens junctions, and gain of expression of the intermediate filament protein vimentin. A number of gene regulatory proteins orchestrating these events have been identified, including Slug, Snail and Twist, which are known to repress E-cadherin expression (2). These in turn can be activated by intracellular signalling pathways, notably the TGFβ-induced Smad pathway and the MAP kinase cascade (3).

In recent years, a rapidly growing number of studies have implicated EMT or EMT-like events in the development of carcinomas, although this matter is still the subject of considerable controversy (3). Proponents of EMT as a mechanism for carcinogenesis point to the loss of E-cadherin and gain of vimentin expression seen in cells at the invasive front of epithelial tumours (4) and the frequent acquisition of malignant properties in cells where EMT has been experimentally induced (5,6). Moreover, recent findings indicate that cells generated by EMT share characteristics with cancer stem cells (7).

E-cadherin has long been regarded as a crucial target of EMT-induced changes in gene expression. In epithelial cells, E-cadherin is the transmembrane protein responsible for homophilic binding between structures of adherens junctions of neighbouring cells. On its cytoplasmic side, E-cadherin is associated with the actin cytoskeleton by interaction with β- or γ-catenin. It is generally believed that these adaptor molecules bind to actin filaments by interaction with α-catenin (8), although the precise role for this protein has been vigorously debated in recent years (9). E-cadherin has also been identified as a tumour suppressor protein, based on the observations that its expression often is lost during carcinogenesis and forced re-expression has been seen to reverse malignant properties of carcinoma cells (10). Loss of E-cadherin expression is also generally viewed as a decisive event in EMT where it is widely assumed to be the direct cause of cell-cell detachment (1).

We have previously (11,12) described the induction of EMT in a cellular model for mammary carcinogenesis caused by c-erbB2 (HER2), an oncogenic receptor tyrosine kinase (RTK) over-expressed in a subset of mammary cancers. This overexpression is thought to cause activation of c-erbB2 by ligand-independent homodimerisation (contrasting with its normal function in ligand-induced heterodimerisation with other EGF receptor family members) (13). In our model, high c-erbB2 signalling is induced in an immortalised human luminal mammary epithelial cell line [HB2, originally developed in the lab of Dr Joyce Taylor-Papadimitriou (14)] by means of a hybrid RTK, ‘trk-neu’. This hybrid consists of the transmembrane and cytoplasmic domains of c-erbB2 fused to the extracellular domain of the trkA nerve growth factor (NGF) receptor (15). Expression of this construct allows signalling from a homodimer of c-erbB2 to be induced by treatment of the cells with NGF. In earlier studies we observed that prolonged induction of c-erbB2 signalling in HB2 cells stably expressing the trk-neu hybrid receptor caused irreversible EMT (5). Furthermore, and as observed by other researchers (11,16,17), the progression of EMT was significantly delayed when cells were grown at high density, a finding which is in good accordance with clinical studies preferentially detecting EMT markers at the invasive front of a tumour, where cell density is at a minimum (18,19). The phenomenon of density-dependent inhibition of EMT certainly merits further investigation as it offers insight into a mechanism whereby cancer cell invasiveness might be counteracted. It was also observed that cells which had recently scattered as a consequence of c-erbB2 signalling (i.e., commenced the EMT process) still expressed surface-bound E-cadherin (11), calling into question the key causal role ascribed to E-cadherin loss in EMT. Here we have further analysed these phenomena by following the progression of EMT while ectopically expressing wild-type or dominant-negative E-cadherin in trk-neu expressing HB2 cells. EMT was not impeded, nor were fibroblastic cells isolated after EMT affected in their morphology by the presence of ectopically expressed wild-type E-cadherin, suggesting that downregulation of E-cadherin expression is indeed not required for the progression of EMT. In an attempt to elucidate whether density-dependent inhibition of EMT was mediated by intercellular E-cadherin engagement, a dominant-negative E-cadherin mutant deficient in adhesiveness was expressed. While cell-cell adhesion was weakened, the mutant failed to promote c-erbB2-induced EMT at high cell density, suggesting that E-cadherin signalling does not account for density-dependent inhibition of EMT.

## Materials and methods

### Materials

Dulbecco’s modified Eagle’s medium (DMEM), fetal bovine serum (FBS) zeocin, blasticdin S, Geneticin, and the plasmid vectors pcDNA3.1- and pcDNA5/TO were from Invitrogen (Carlsbad, CA, USA). Insulin, hydrocortisone, doxycycline and Triton X-100 were from Sigma (St. Louis, MO, USA). The monoclonal antibody (mAb) HECD-1 against E-cadherin was from Takara Bio (Shiga, Japan). The mAb Vim3B4 against vimentin was from Dakopatts AB (Älvsjö, Sweden) and R-phycoerythrin-labeled goat anti-mouse antibody was from Electra-Box Diagnostica (Tyresö, Sweden). Antibodies against β-catenin, Slug and ZO-1 were from Cell Signalling Technology (Danvers, MA, USA). The γ-catenin antibody (Transduction Laboratories, Lexington, KY, USA) was a kind gift from Professor Mikael Nilsson, University of Gothenburg. Antibodies against α-tubulin and human fibronectin were from Sigma. Restriction endonucleases were from Fermentas (Pittsburgh, PA, USA). The 2.5S nerve growth factor (NGF) from mouse submaxillary gland was purchased from Promega (Madison, WI, USA). The pIRES2-GFP plasmid was from Clontech (Mountain View, CA, USA) and the pcDNA3.1- and pcDNA5/TO plasmids were from Invitrogen. Full-length human E-cadherin cDNA in pBluescript had previously been obtained as a gift from the late Professor Margaret Wheelock.

### Cell culture

MDA-MB-468 cells (no. HTB-132, ATCC, Manassas, VA, USA) were grown in DMEM containing 10% FBS. Tr-ep cells were grown in DMEM with 10% FBS 5 *μ*g/ml hydrocortisone, 10 *μ*g/ml insulin, 5 *μ*g/ml zeocin and 10 *μ*g/ml blasticidin S. Transfectants carrying IRES-GFP constructs also had 0.5 mg/ml Geneticin added to the medium. Cells were grown at 37°C and 5% CO_2_.

### cDNA constructs

Expression constructs were based on pcDNA3.1neo/TO-IRES-GFP, a vector constructed in the lab by first combining the commercially available vectors pcDNA3.1- (neo resistance) and pcDNA5/TO (zeocin resistance) using cleavage with *Mlu*I and *Xma*I in both plasmids and combining the tetracycline operator-containing fragment from pcDNA5/TO and the neo resistance-containing fragment from pcDNA3.1-. The resulting plasmid, pcDNA3.1neo/TO, was then further modified by inserting the IRES-GFP sequence from pIRES2-GFP as an *Xho*I-*Xba*I fragment, and then deleting duplicated polylinker restriction sites by site-directed muta-genesis. Human E-cadherin cDNA was then introduced into this vector to yield pcDNA3.1neo/TO/E-cadherin-IRES-GFP. Further site-directed mutagenesis was used to obtain the WV156-157AA (WV) E-cadherin mutant. Transformants were screened for the introduction of a *Nae*I site and selected clones were sequenced through the coding region.

### Transfections

Expression constructs were introduced by calcium phosphate co-precipitation using standard techniques (20). Desired clones were identified by fluorescence microscopy following brief (24 h) doxycycline (dox) treatment and then recovered by detachment in cloning cylinders. Further characterisation was performed by measuring GFP fluorescence and binding of E-cadherin mAb HECD-1 in flow cytometry.

### Flow cytometry

Flow cytometric measurements were performed as described (11) with the following modifications: cells were detached with trypsin-EDTA, and where permeabilisation was required, cells were treated with 0.5% Triton X-100 (ice, 10 min). It should be noted that unless otherwise indicated, non-permeabilised cells were used for analysis of E-cadherin expression in order to exclusively detect plasma membrane-bound E-cadherin. mAb HECD-1 (10 *μ*g/ml) was used for detection of E-cadherin and the mAb Vim3B4 (1:100 dilution) was used to probe for vimentin. R-phycoerythrin-labeled goat anti-mouse antibody was used as a secondary reagent. Dox-treated (i.e., GFP-expressing) cells incubated with secondary antibody only were used as controls for compensation of leakage of GFP fluorescence into the FL2 channel used to detect R-phycoerythrin fluorescence. In some experiments, allophycocyanin-labeled goat anti-mouse antibody was used as a secondary reagent; here, compensation was not required.

### EMT assays

Cells (7×10^4^) were seeded into T75 cell culture flasks and grown under the following conditions: no treatment, NGF (50 ng/ml), or NGF + dox (1 *μ*g/ml). In order to ensure an even level of dox-induced expression, additional dox corresponding to 0.5 *μ*g/ml was supplied every second day. When cells reached 50% confluence (typically after 3–4 days), they were trypsinised and a new passage of cells was seeded as described above. The remainder of the cells were analysed for E-cadherin and vimentin expression in flow cytometry. This procedure was repeated for at least four passages.

The significance of the changes in E-cadherin expression was analysed as follows: a linear model was used to analyse the data. Logarithmic fluorescence values were used as the dependent variable and passage, treatment and replicate were regarded as factors. An interaction term between passage and treatment was used. Tests of difference for each time between the respective treatments and control cells were performed. When evaluating the percentage of vimentin-positive cells, the cutoff fluorescence level for defining cells as positive was set to the level where 99.5% of untreated cells were excluded.

### Isolation of fibroblastic clones

From TrE-ep1 cells passaged at low density in the presence of NGF and dox as described above for EMT assays, fibroblastic clones were prepared by plating at 300–1,000 cells per 9-cm dish, growing in the continued presence of NGF and dox, and isolating individual colonies with fibroblastoid cell morphology using cloning cylinders.

### Microscopy

Cells grown on glass coverslips in 24-well plates were fixed in 4% paraformaldehyde in PBS for 20 min at room temperature (RT), and where permeabilisation was required, cells were treated with 0,5% Triton X-100 for 5 min on ice (in order to restrict detection of E-cadherin to surface-bound molecules, only non-permeabilised cells were used in these analyses). Cells were further blocked in 20% FBS in PBS for 20 min in RT, incubated with primary antibody (diluted in 5% FBS in PBS) for 1 h at RT, washed 2×5 min in PBS, incubated with secondary TRITC-conjugated antibody diluted in 5% FBS in PBS, washed 2×5 min in PBS and mounted with ProLong Antifade Gold (Molecular Probes, Eugene, OR, USA). Fluoromicrographs were taken in a Zeiss Axioplan 2 microscope at ×40 magnification (Carl Zeiss, Oberkochen, Germany). At least three different micrographs were evaluated for each condition and representative details were processed with respect to brightness and contrast (consistently between samples) using GraphicConverter (Lemke Software, Peine, Germany).

### Western blot analysis

For whole-cell extract preparation, cells were treated with lysis buffer 150 mM NaCl, 50 mM Tris-HCl (pH 8), 1% Triton X-100 and 1X complete protease inhibitors (Roche, Indianapolis, IN, USA) for 30 min at 4°C. Protein concentrations were determined by using Bio-Rad Protein Assay. A total of 40 *μ*g protein/well was loaded and electrophoresed through a NuPAGE 4 to 12% Bis-Tris sodium dodecyl sulfate-polyacrylamide gel (Invitrogen) and subsequently electroblotted onto a Hybond-P filter (GE Healthcare, Little Chalfont, UK).

### Detergent extraction assay

Cells were trypsinised, dispersed and strained as described for flow cytometry, and then incubated in 0.1 ml of FACS diluent (DMEM, 10% FCS, 0.02% sodium azide) containing 1 *μ*g E-cadherin mAb HECD-1 for 1 h. After three washes with FACS diluent, cells were extracted for 10 min on ice in 500 *μ*l of PBS containing 0.5% Triton X-100. Cells were again washed, incubated with R-phycoerythrin-labeled goat anti-mouse antibody and fluorescence was measured in flow cytometry as described above. The E-cadherin levels were compared with parallel samples stained without detergent extraction.

### Dissociation assays

Cells were grown to confluence in 6-well plates with no treatment, with dox (1 *μ*g/ml) and/or NGF (50 ng/ml) for 2–3 days. Parallel samples were either washed with Puck’s saline containing 0.02% EDTA and detached with trypsin (0.05%), or washed with thermolysin buffer (10 mM HEPES, 142 mM NaCl, 6.7 mM KCl, 0.43 mM NaOH and 1 mM CaCl_2_) and detached with thermolysin (0.5 mg/ml in thermolysin buffer). After mechanical dissociation by passage 4 times through a 23G needle, the total number of cells was counted in the trypsin-treated sample (where E-cadherin is degraded) and the number of ‘particles’, i.e., single cells or cell aggregates, was counted in the thermolysin-treated sample (where E-cadherin is preserved) using a haemocytometer. Changes in the degree of dissociation were expressed as a dissociation index, calculated as DI = (P_t_/C_t_)/(P_u_/C_u_) where P and C represent thermolysin-detached particles and trypsin-detached cells, respectively, and the indices u and t represent untreated and treated cells, respectively.

## Results

### Generation of cells with tetracycline-inducible expression of E-cadherin and GFP

**Figure 1.**
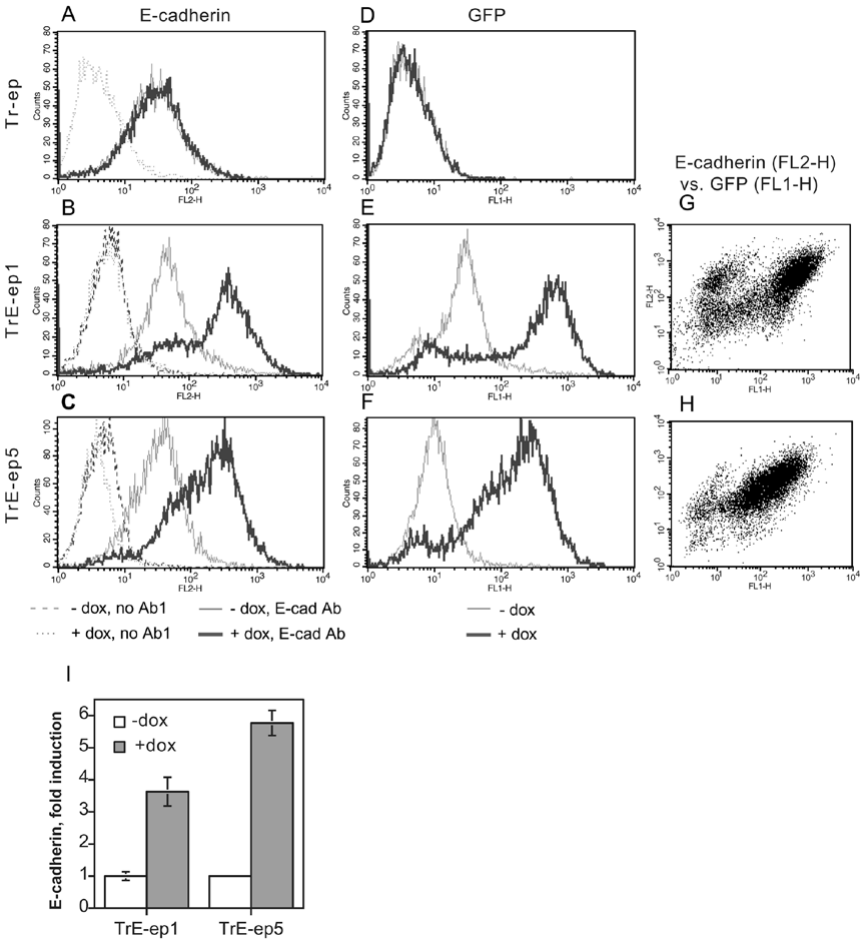
Expression of GFP and E-cadherin in HB2/tnz34 cells expressing the tetracycline repressor only (Tr-ep, A and D) or the tetracycline repressor plus the tetracycline operator-controlled E-cadherin-IRES-GFP construct (TrE-ep1, B, E and G; and TrE-ep5, C, F and H). Expression was analysed by flow cytometry 2 days after ectopic E-cadherin expression was induced by addition of doxycycline (dox) where indicated. E-cadherin was assayed in non-permeabilised cells by the HECD-1 mAb and an RPE-conjugated secondary antibody (FL-2) whereas GFP was measured in the FL-1 channel. (G and H) GFP vs. E-cadherin fluorescence in dox-treated samples. (A–H) Representative data from experiments performed at least three times. (I) Fold induction of E-cadherin expression upon dox treatment, based on mean fluorescence values averaged over at least three independent experiments. Error bars, SEM.

The HB2/tnz34 cell line (5) is derived from the human luminal mammary epithelial cell line HB2 (14) which has been transfected with the hybrid ‘trk-neu’ receptor construct (15), allowing induction of c-erbB2 signalling by means of NGF treatment (see Table I for naming of transfectants and clones). In order to elucidate the role of modulation of E-cadherin expression in c-erbB2 induced EMT, a transfectant clone of HB2/tnz34 stably expressing the tetracycline repressor (Tr-ep) was further transfected with the pcDNA3.1neo/TO/E-cadherin-IRES-GFP plasmid. Using this construct, bicistronic expression of wild-type E-cadherin and GFP can be induced by addition of tetracycline or its analogue, doxycycline (dox). Stable clones from this transfection were selected and analysed for dox-induced E-cadherin and GFP expression. Two clones, designated TrE-ep1 and TrE-ep5, were chosen for further analysis (Fig. 1). The majority of cells in these clones overexpressed E-cadherin upon dox treatment with concomitant GFP expression. There was a good correlation between GFP and E-cadherin expression levels (Fig. 1G–H). In a minority of cells, E-cadherin overexpression was seen without GFP fluorescence, possibly suggesting a longer half-life for E-cadherin in the induced cells compared to GFP. The reverse expression pattern, i.e., GFP expression without E-cadherin overexpression, was not seen. Hence, practically all cells expressing GFP could be assumed to overexpress E-cadherin.

### Ectopic expression of E-cadherin does not affect c-erbB2-induced EMT

**Figure 2.**
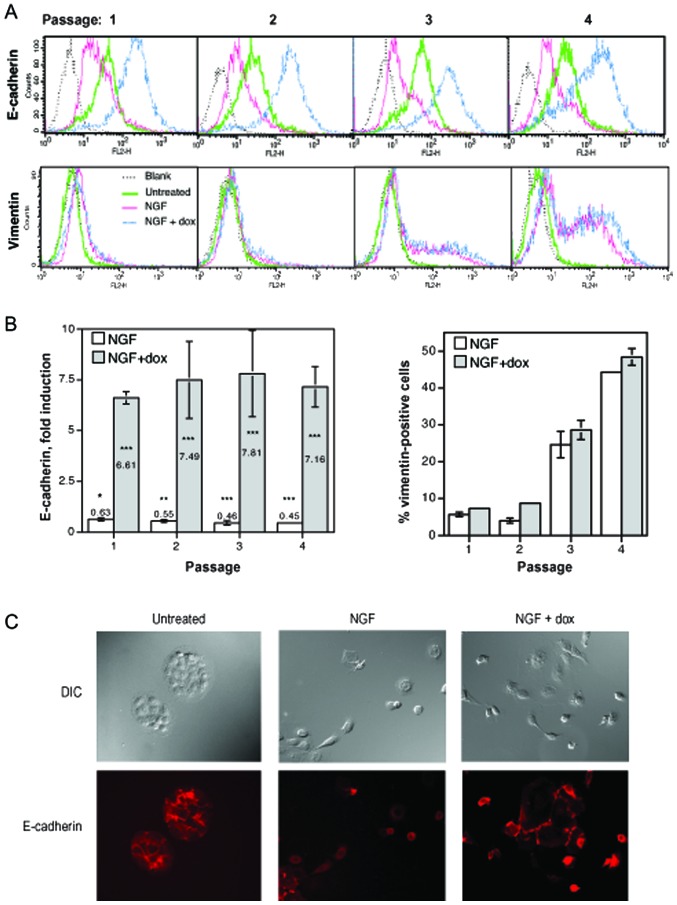
c-erbB2-induced EMT in cells with and without ectopic E-cadherin expression. (A) Representative histograms from flow cytometry measurements of E-cadherin and vimentin expression in TrE-ep5 cells passaged at low density as outlined under EMT assays in Materials and methods with NGF (to induce c-erbB2 signalling), dox (to induce E-cadherin expression) + NGF, or untreated. (B) Summary of two independent EMT assay experiments. Fold induction of E-cadherin was calculated as the ratio between the mean fluorescence intensity of the sample cells (white bars, NGF; grey bars, NGF + dox) relative to that of untreated cells. The statistical significance of the changes in E-cadherin expression relative to control cells was analysed as described in Materials and methods and is denoted by the asterisks in the graph (^*^p<0.05; ^**^p<0.02; ^***^p<0.01). Vimentin-positive cells were defined as described in Materials and methods. The experiments shown in (A) and (B) have also been performed with TrE-ep1 cells, with similar results. (C) Representative micrographs showing DIC and E-cadherin immunofluorescence in TrE-ep5 cells grown at low density for 5 days in the presence of NGF, NGF + dox or untreated. Note that in all experiments, E-cadherin was measured in nonpermeabilised cells in order to exclusively detect the plasma membrane-associated fraction of this protein.

We next used the transfectant clone TrE-ep5 in order to study the possible influence of ectopic E-cadherin expression during the process of c-erbB2-induced EMT. Cells were grown at low density for four passages with c-erbB2 signalling (NGF treatment), with c-erbB2 signalling and ectopic E-cadherin expression (NGF and dox treatment) or untreated. For each passage, EMT was monitored by analysis of E-cadherin and vimentin expression as outlined under EMT assays in Materials and methods. As expected, c-erbB2 signalling alone caused a reduction in E-cadherin expression and a gradual increase in the appearance of vimentin-positive cells (Fig. 2A and B, NGF). Combining c-erbB2 signalling with dox treatment caused a substantial increase in E-cadherin expression (also as expected), but the high level of E-cadherin did not affect the increase in vimentin-positive cells (Fig. 2A and B, NGF + dox) which was highly similar to that seen with c-erbB2 signalling alone. Ectopic E-cadherin expression thus did not appear to prevent the process of c-erbB2-induced EMT.

We also used immunofluorescence microscopy to analyse TrE-ep5 cells for E-cadherin surface expression (Fig. 2C). Treatments with NGF or NGF + dox had been performed for 5 days in this experiment, which is the earliest time point at which significant scattering can be observed. Here, NGF-treated and NGF + dox-treated as well as untreated cells all expressed detectable levels of E-cadherin. Importantly, expression was evident also in scattering cells, confirming that cell-cell dissociation was not prevented by E-cadherin. At later passages, scattered cells treated only with NGF largely lost E-cadherin expression indicating a transition to the fully mesenchymal-like phenotype (data not shown).

**Figure 3.**
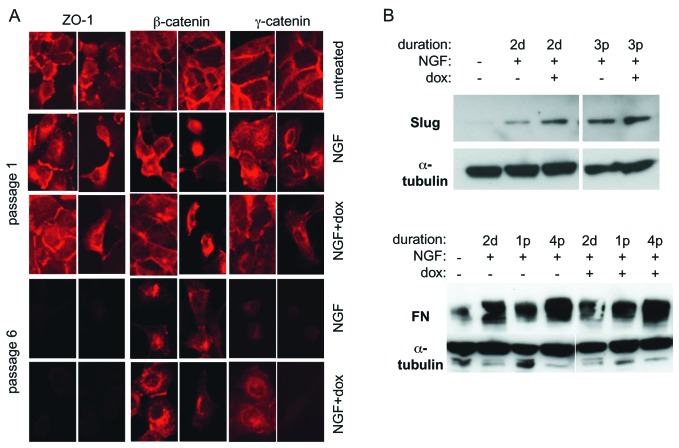
Expression and localisation of EMT markers during the progression of c-erbB2-induced EMT. (A) Immunofluorescence micrographs of permeabilised TrE-ep5 cells stained with antibodies to ZO-1, β-catenin and γ-catenin. (B) Western blot analysis of the expression of EMT markers Slug and fibronectin (FN). Cells were treated with NGF or NGF + dox for the indicated durations (d, days; p, passages) or left untreated before lysis.

In order to further study the possible influence of E-cadherin overexpression on EMT in this system, the expression of EMT markers was analysed by immunofluorescence microscopy and western blot analysis. Fig. 3A shows expression and subcellular localisation of the tight junction-associated protein ZO-1 as well as the E-cadherin-associated cytoskeletal linker proteins β- and γ-catenin. ZO-1 expression was maintained in recently scattered cells but subsequently lost. β-catenin showed the redistribution from plasma membrane-associated to cytoplasmic and nuclear/perinuclear localisation typical of EMT, both when treated with NGF and NGF + dox. γ-catenin was also relocalised but with a concomitant sharp decrease in expression level at later passages. These data suggest that ZO-1 and γ-catenin, like E-cadherin, are not lost until after the initial cell-cell detachment phase of EMT. In no case did dox-induced E-cadherin expression influence these changes.

Western blot analysis showed an early (2 days after induction of c-erbB2 signalling) upregulation of the EMT markers Slug and fibronectin (Fig. 3B) which persisted in subsequent passages. Again, E-cadherin expression did not prevent these EMT-associated changes. Together, these data indicate that ectopic expression of E-cadherin is not a significant obstacle to EMT.

**Figure 4.**
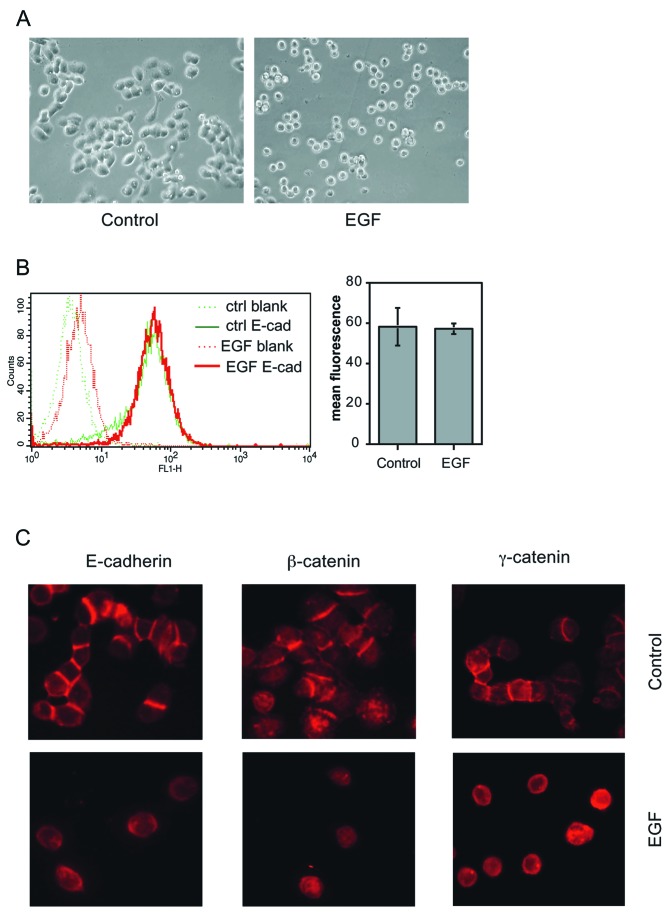
Characterisation of EGF-induced cell scattering in the mammary carcinoma cell line MDA-MB-468. In all experiments, cells were grown without EGF until spread and engaged in cell-cell contact. EGF treatment was then performed overnight and at a concentration of 50 ng/ml. (A) Morphology of untreated and EGF-treated MDA-MB-468 cells. (B) Analysis of E-cadherin surface expression in control and EGF-treated MDA-MB-468 cells using flow cytometry of non-permeabilised cells. The left-hand panel shows a histogram from one of the measurements and the right-hand panel shows mean fluorescence intensity (blank subtracted) averaged for two experiments. (C) Immunofluorescence micrographs showing untreated and EGF-treated MDA-MB-468 cells stained with antibodies to E-cadherin (non-permeabilised cells), β-catenin and γ-catenin.

In order to verify that EMT-associated cell scattering without reduction in cell surface E-cadherin expression can occur also in other cell systems, we examined the early stages of EMT in the mammary carcinoma cell line MDA-MB-468. This cell line has previously been shown to undergo EMT upon EGF treatment with only partial reduction in E-cadherin expression (21,22). We found that overnight treatment of MDA-MB-468 cells with EGF caused extensive cell-cell dissociation (Fig. 4A). Strikingly, cell surface expression of E-cadherin (as measured by flow cytometry of unpermeabilised cells) was virtually unchanged by EGF at this stage (Fig. 4B). This finding was confirmed by immunofluorescence microscopy, which furthermore showed that β-and γ-catenin expression was also retained (Fig. 4C). These data suggest that cell scattering without the prerequisite for downregulation of surface E-cadherin expression may be a common feature in the early steps of EMT caused by EGFR family receptor activation.

### Properties of fibroblastic cells isolated after c-erbB2-induced EMT in E-cadherin-overexpressing cells

**Figure 5.**
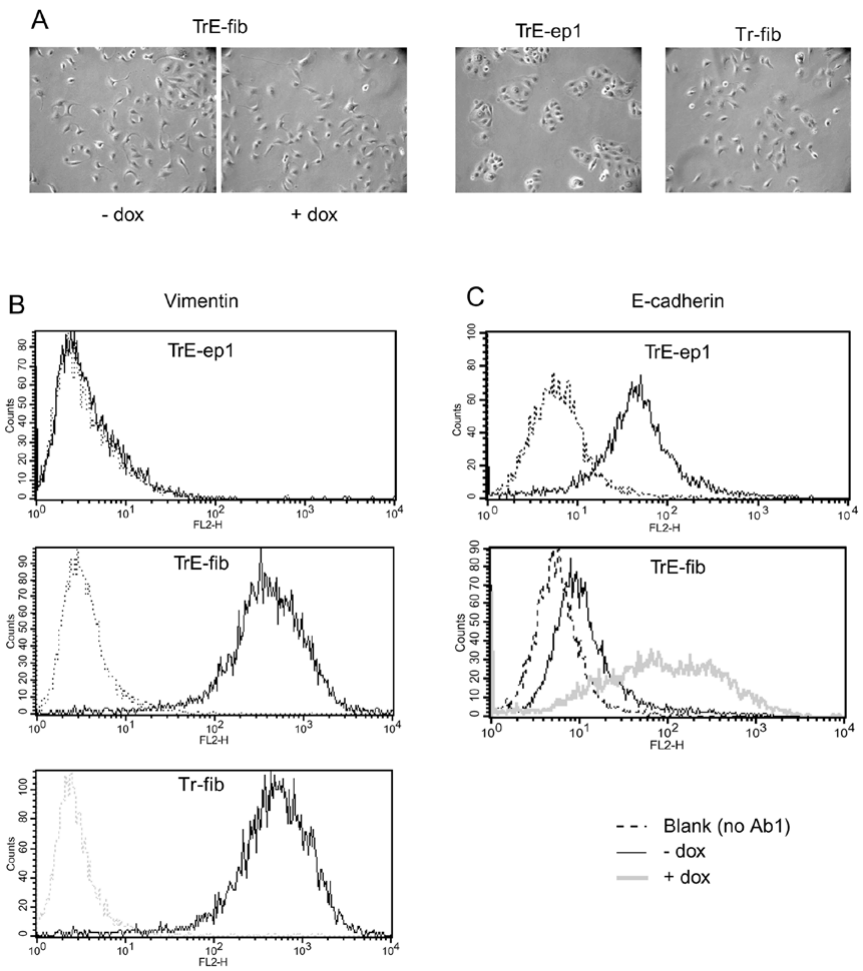
Morphology and expression of vimentin and E-cadherin in the fibroblastic clone TrE-fib isolated after c-erbB2-induced EMT with concomitant induced expression of E-cadherin. (A) Micrographs showing morphology of TrE-fib cells with and without dox treatment for one week (prolonged treatment showed the same results). TrE-ep1 and Tr-fib cells (the latter generated by c-erbB2-induced EMT of Tr-ep cells, i. e. lacking the E-cadherin-IRES-GFP construct) (11,12), are shown for comparison. (B) Expression of the mesenchymal marker vimentin in TrE-ep1, TrE-fib and Tr-fib cells. (C) Expression of E-cadherin with and without dox treatment for two days in TrE-ep1 cells and TrE-fib cells. Expression levels in (B) and (C) were measured by flow cytometry (in B following permeabilisation by Triton X-100 treatment).

In order to further study the properties of cells having undergone EMT in the presence of E-cadherin, fibroblastic clones were isolated from a population of TrE-ep1 cells that had been treated with dox and NGF for several passages. One clone, named TrE-fib, was selected for further study, as its induced E-cadherin expression was comparable to that of parental epithelial cells (see below). TrE-fib cells showed a fibroblastoid morphology and strong vimentin expression (Fig. 5A and B) typical of HB2 cells after EMT (compare with Tr-fib cells, fibroblastic cells obtained from Tr-ep cells by c-erbB2-induced EMT but lacking the E-cadherin-IRES-GFP construct, in Fig. 5A and B). E-cadherin expression was negligible in dox-untreated cells, indicating that the endogenous *CDH1* gene had been silenced (Fig. 5C). These properties did not change following prolonged culture without NGF or dox (data not shown), suggesting an irreversible phenotypic conversion, in line with previous results on EMT in HB2 cells (11). Upon dox treatment, E-cadherin expression was readily induced (Fig. 5C). However, no changes in cell morphology were seen following E-cadherin induction in this clone (Fig. 5A).

### E-cadherin ectopically expressed in fibroblastic cells after EMT is poorly attached to the cytoskeleton

**Figure 6.**
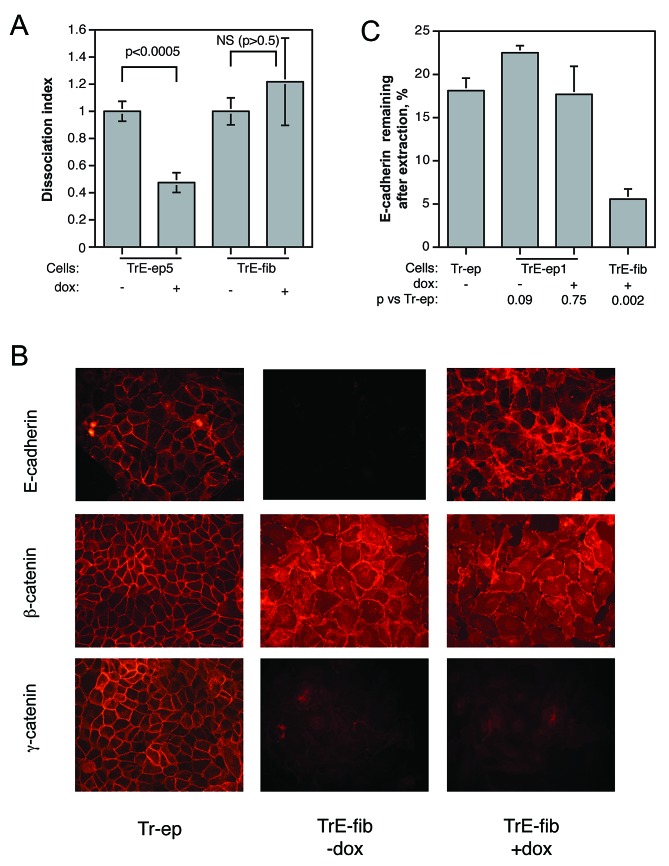
Characterisation of fibroblastic cells with respect to cell-cell adhesion and localisation and cytoskeletal attachment of E-cadherin. (A) Influence of forced E-cadherin expression on cell-cell adhesion, as measured by dissociation assay, in epithelial TrE-ep5 and fibroblastic TrE-fib cells (p-values obtained by Student’s t-test; NS, not significant). (B) Immunofluorescence micrographs showing localisation of E-cadherin, β-catenin and γ-catenin in Tr-ep cells and in TrE-fib cells with and without dox treatment. For analysis of E-cadherin, cells were not permeabilised before staining; thus, only cell surface-bound E-cadherin is visualised. (C) Percentage of E-cadherin detected after extraction of membrane lipids with Triton X-100 in Tr-ep, TrE-ep1 and TrE-fib cells with and without dox treatment as indicated. n, number of independent experiments; p, p-value in Student’s t-test for comparison with results for Tr-ep cells. Error bars, SEM.

The apparent lack of effect of forced E-cadherin expression on the phenotype of the fibroblastic cells emerging after EMT raised the question whether E-cadherin was functional as a cell adhesion molecule under these circumstances. We therefore performed dissociation assays on cells from confluent layers of TrE-ep5 and TrE-fib cells in the presence or absence of dox. In striking contrast to the restoring effect on cell-cell adhesion seen in dox-treated epithelial cells, dox-induced E-cadherin expression in confluent fibroblastic TrE-fib cells failed to influence intercellular adhesion (Fig. 6A). This result strengthened the notion that the function of E-cadherin was impaired in the fibroblastic cells. We therefore sought to elucidate the cause of this impairment. Immunofluorescence microscopy of non-permeablilised, dox-treated TrE-fib cells showed that E-cadherin was predominantly present at cell-cell contacts in a manner roughly similar to that seen in parental epithelial cells, although diffuse staining distributed over the cell surface was also observed (Fig. 6B). This suggests that gross abnormalities in the localisation of E-cadherin were not a cause of malfunction.

Another mechanism by which E-cadherin function could be disrupted is loss of cytoskeletal attachment. The cytoskeletal linker proteins β-catenin and γ-catenin were assayed in immunofluorescence microscopy (Fig. 6B). β-catenin, as expected, showed increased cytoplasmic and nuclear staining in the TrE-fib cells compared to control Tr-ep cells, but also significant amounts close to the plasma membrane. In contrast, γ-catenin expression was strongly decreased with complete relocalisation to the cytoplasm and nucleus. These EMT-induced changes in β- and γ-catenin expression and localisation were not affected by ectopic E-cadherin expression (i.e., dox treatment). We further examined the role of E-cadherin cytoskeletal anchorage by measuring the percentage of surface-bound E-cadherin still remaining after extraction of membrane lipids by Triton X-100 treatment. This procedure should remove cell surface proteins attached only via interactions between the transmembrane domains and the lipid bilayer, whereas proteins bound to the cytoskeleton should be preferentially retained. As shown in Fig. 6C, the E-cadherin ectopically expressed in fibroblastic cells isolated after EMT was much easier to extract than E-cadherin in parental epithelial cells. These results suggest that following EMT, ectopically expressed E-cadherin has a poor cytoskeletal anchorage, offering a possible explanation for its lack of effect on cell phenotype.

### Expression of dominant-negative E-cadherin fails to expedite EMT at high cell density

**Figure 7.**
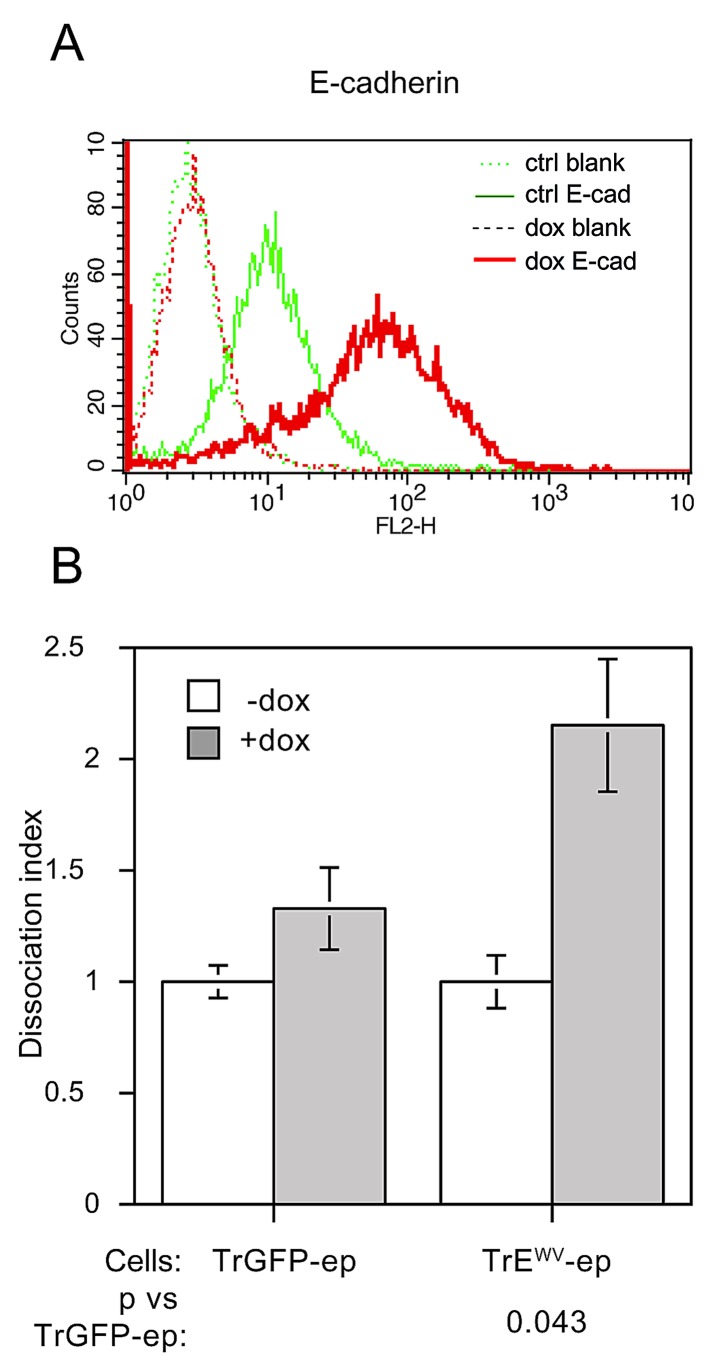
Properties of Tr-ep cells expressing dominant-negative E-cadherin. (A) Dox-induced expression of the E-cadherin ‘WV’ mutant construct, detected as an increase in total E-cadherin levels (using mAb HECD-1 staining of non-permeabilised cells), in flow cytometry. (B) Effects of E-cadherin mutant expression on cell-cell adhesion as measured in dissociation assay (see Materials and methods for details). TrGFP-ep cells, Tr-ep cells expressing GFP only from the TO/IRES-GFP construct, were used as control. p, p-value in Student’s t-test for comparison with TrGFP-ep cells. Error bars, SEM.

The density-dependent inhibition of EMT which has previously been observed by us (11) and others (16,17) indicates that cell-cell contact elicits intracellular signalling events which impede the EMT process. The autoregulatory properties of E-cadherin expression described by Conacci-Sorrell *et al* (23) suggest that E-cadherin itself may be a sensor for the degree of cell-contact experienced by a cell. If this were indeed the case, interfering with the proper function of E-cadherin as an adhesion molecule would abrogate density-dependent inhibition of EMT and thus allow EMT to occur at high cell density. In order to test this hypothesis, we used the extracellular domain mutant WV156–157AA (WV), a well-characterised mutant in which the adhesiveness of E-cadherin is abolished (24). This WV mutant was also expressed as a tetracycline-regulated bicistronic IRES-GFP construct in Tr-ep cells. Stable transfectants harbouring this construct were generated and the clone TrE^WV^-ep was selected for further study. As shown in Fig. 7A, robust expression of the mutant construct was induced upon dox treatment. A significant decrease in cell-cell adhesion, as measured by cell dissociation assay (Fig. 7B) was observed upon induction of mutant expression.

**Figure 8.**
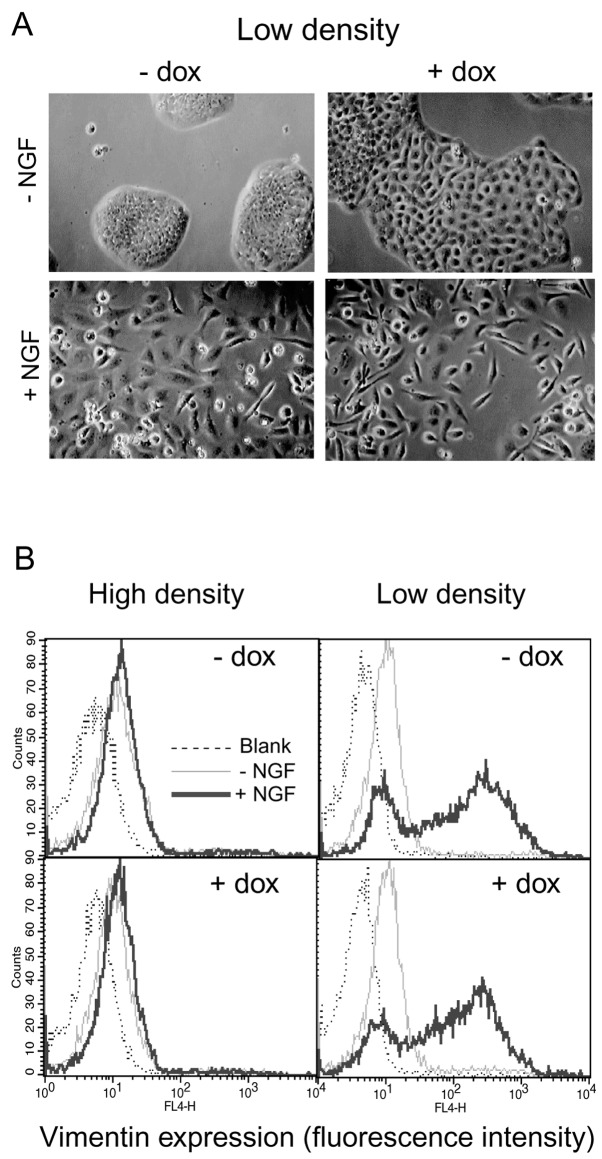
Expression of mutant E-cadherin fails to promote c-erbB2-induced EMT at high cell density. (A) Photomicrographs of TrE^WV^-ep cells grown at low density for two passages with or without dox and NGF treatment as indicated. Note that all micrographs are taken at the same magnification. (B) Expression of the mesenchymal marker vimentin in TrE^WV^-ep cells after 4 passages in the absence or presence of NGF and/or dox at low or high cell density, as measured by flow cytometry.

When TrE^WV^-ep cells were grown at low density, dox-induced mutant expression without concomitant c-erbB2 signalling caused some morphological changes in the cells: although still largely epithelial in appearance, the dox-treated cells occupied a much larger surface area per cell and the cell-cell borders were more clearly visible (Fig. 8A, top row). At this low density, NGF-induced c-erbB2 signalling caused the appearance of fibroblast-like cells both with and without mutant expression (Fig. 8A, lower panel). However, c-erbB2-induced EMT at high cell density did not appear to be derepressed upon expression of the non-adhesive E-cadherin. Vimentin expression was virtually absent in cells grown at high density irrespective of treatment with NGF, dox or both, whereas NGF treatment caused the appearance of vimentin-positive cells at low cell density, equally well with or without dox-induced mutant expression (Fig. 8B). These data do not support a role for E-cadherin as a sensor for cell-cell contact in density-dependent inhibition of EMT.

## Discussion

The possible role of EMT as a mechanism for carcinogenesis, particularly in the generation of invasive, metastatic and treatment-resistant cells, has been the subject of intense study for the last few years and remarkable advances in our understanding of these phenomena have been made (1). However, comparatively few recent studies have been devoted to the interplay between cell-cell contact/adhesion and EMT. Loss of E-cadherin expression is widely seen as one of the most crucial and defining events in the progress of EMT (25), and since EMT is characterised by loss of cell-cell adhesion, it is intuitively easy to assume that downregulation of E-cadherin expression is required for EMT to occur. This notion has been reinforced by numerous studies where E-cadherin has been re-expressed in carcinoma cells yielding cells with variable degrees of restoration of epithelial phenotype and non-malignant behaviour (26). In contrast, the necessity of loss of E-cadherin expression for the actual progress of EMT has not been extensively studied in a rigorous manner. Even less attention has been paid to the mechanisms underlying density-dependent inhibition of EMT, a highly interesting phenomenon noted in several studies but rarely elaborated upon.

In an earlier publication (11), we noted that cells apparently undergoing the first scattering phase in c-erbB2-induced EMT still expressed E-cadherin, thus raising the question of whether loss of E-cadherin is a cause or a consequence of EMT. In the present study, we have addressed this question by creating cells which are capable both of NGF-inducible c-erbB2 homodimer signalling and dox-inducible E-cadherin expression. The E-cadherin ectopically expressed in these cells consistently failed to prevent c-erbB2-induced EMT from occurring (Fig. 2), strengthening the hypothesis that the presence of E-cadherin is not an impediment to the progression of EMT.

Our observation of extensive EGF-induced cell scattering with unaltered E-cadherin surface expression in the MDA-MB-468 cell line (Fig. 4) strengthens the notion that surface-bound E-cadherin can be rendered incompetent at maintaining cell-cell adhesion and that EMT-associated cell scattering without requirement for prior downregulation of E-cadherin is not a cell line-specific event. Further investigation into this phenomenon and its possible occurrence in other instances of EMT is clearly merited.

The finding that cell-cell dissociation occurs in spite of the robust expression of a major cell-cell adhesion molecule suggests that the adhesive function of E-cadherin becomes compromised during EMT, before expression is lost. Several mechanisms could account for such an inactivation, including weakening of the cytoskeletal attachment or increased turnover or endocytosis of E-cadherin. Influence from cytoskeletal rearrangements on E-cadherin function is suggested by our results with fibroblastic cells: here, E-cadherin expression had no effect on cell-cell adhesion and the expressed E-cadherin was much more readily extracted from the plasma membrane than in the epithelial cells (Fig. 6). It is possible that some component of the linkage between E-cadherin and the cytoskeleton was inactivated in the fibroblastic cells, e.g. by phosphorylation or ubiquitinylation of E-cadherin or β-catenin. However, we have failed to detect changes in β-catenin phosphorylation using site-specific mAbs (data not shown) although the interpretation of such data is complicated by the non-synchronous nature of c-erbB2-induced EMT in the system studied.

Another cytoskeletal mechanism, more supported by our data, could be envisioned where the combined influences of c-erbB2 and low cell density trigger extensive cytoskeletal rearrangements which make the cells unable to support strong cell-cell adhesion, even in the presence of E-cadherin expression. This suggestion is supported by previous data showing a dramatic destabilisation of the cortical cytoskeleton following c-erbB2 activation in HB2 cells (27).

It is interesting to note that onset of cell scattering prior to E-cadherin downregulation in EMT has been observed in another system (28); there, E-cadherin internalisation was offered as an explanation (29). In our case, net removal of E-cadherin from the plasma membrane is highly unlikely to be the cause of impaired cell-cell adhesion as the measurements of E-cadherin expression were made by flow cytometry of non-permeabilised cells, which only detects surface-bound antigens, although adherens junction destabilisation by increased E-cadherin recycling cannot be excluded. For instance, the combination of Par3 downregulation and c-erbB2 signalling caused invasive and metastatic behaviour in mammary epithelial cells which was attributed to increased F-actin and E-cadherin turnover (30). Other studies have also reported the persistence of E-cadherin expression during EMT (22,31,32), but without direct reference to cell scattering. It has even been suggested that surface expression of E-cadherin promotes EMT-inducing β-catenin signalling (33). Very recently, Hollestelle *et al* (34) have showed a lack of consistent correlation between E-cadherin loss and expression of EMT markers in a survey of 38 breast cancer cell lines as well as in clinical tumour samples. In the same study, restoring E-cadherin expression failed to influence the mesenchymal-like phenotype of E-cadherin-negative cell lines. While not addressing the role of E-cadherin expression during the actual EMT process, those data strongly support the conclusions from the present study.

The other hypothesis tested here concerns the density-dependent inhibition of EMT observed earlier by us and several other researchers. This is a highly interesting phenomenon, especially in the light of the proposed significance of EMT in cancer progression: if invasion and metastasis indeed are dependent on EMT-like phenomena, then a mechanism which inhibits EMT would be of great potential value in combating cancer cell dissemination. A key question in elucidating the signalling pathway responsible for density-dependent EMT inhibition regards the nature of the sensor for cell cell-contact. E-cadherin itself has recently been identified as a mechanosensor (35) and the findings by Conacci-Sorrell *et al* (23) suggest that E-cadherin engagement is important for the maintenance of its own expression. We therefore tested the influence of expression of a dominant-negative E-cadherin mutant on our system. According to our working hypothesis, that E-cadherin engagement suppresses EMT at high cell density, the expression of non-adhesive E-cadherin mutant would relieve this suppression. Although we found a significant reduction in cell-cell adhesion upon expression of this mutant, c-erbB2-induced EMT was still inhibited at high cell density as far as could be measured in our assays. The residual cell-cell adhesion seen upon expression of the E-cadherin mutant could be attributed to cell-cell adhesion molecules other than E-cadherin, but our data cannot rule out an incomplete inactivation of E-cadherin. This should also be kept in mind when interpreting the results. An alternative mechanism for density-dependent inhibition of EMT was found to operate in mouse mammary epithelial cells, which underwent EMT upon overexpression of matrix metalloproteinase 3 (MMP-3); in these cells, the cytoskeletal effects of cell crowding seemed to serve as an anti-EMT signal, as limiting the cell area by growing single cells on a micropatterned substrate was sufficient to prevent MMP-3- (but not TGF-β-) induced EMT (36). It is possible that cytoskeletal effects are more important for regulation of EMT in our system as well. In summary, our results indicate that E-cadherin does not appear to have a key role in preventing c-erbB2-induced EMT, either as a physical obstacle to cell-cell separation at low density or as an mediator of density-dependent inhibition of EMT in confluent cells.

## Figures and Tables

**Table I.** Names and descriptions of cell lines and subclones used in the study.

Cell line	Description
HB2	Cell line established from human luminal mammary epithelial cells (14)
HB2/tnz34	HB2 cells stably transfected with the trk-neu hybrid receptor (5)
Tr-ep	HB2/tnz34 cells stably transfected with the tetracycline repressor; epithelial morphology
Tr-fib	Tr-ep cells having undergone EMT as a result of c-erbB2 signalling (NGF treatment); fibroblastoid morphology
TrE-ep1 TrE-ep5	Tr-ep cells stably expressing wild-type E-cadherin and GFP under the tetracycline operator; epithelial morphology
TrE-fib	Clone of TrE-ep1 cells having undergone EMT as a result of c-erbB2 signalling (NGF treatment) with concomitant ectopic E-cadherin expression (dox treatment); fibroblastoid morphology
TrE^WV^-ep	Tr-ep cells stably expressing the E-cadherin WV156-157AA mutant and GFP under the tetracycline operator; epithelial morphology
TrGFP-ep	Tr-ep cells stably expressing the ‘empty’ IRES-GFP construct under the tetracycline operator; epithelial morphology
